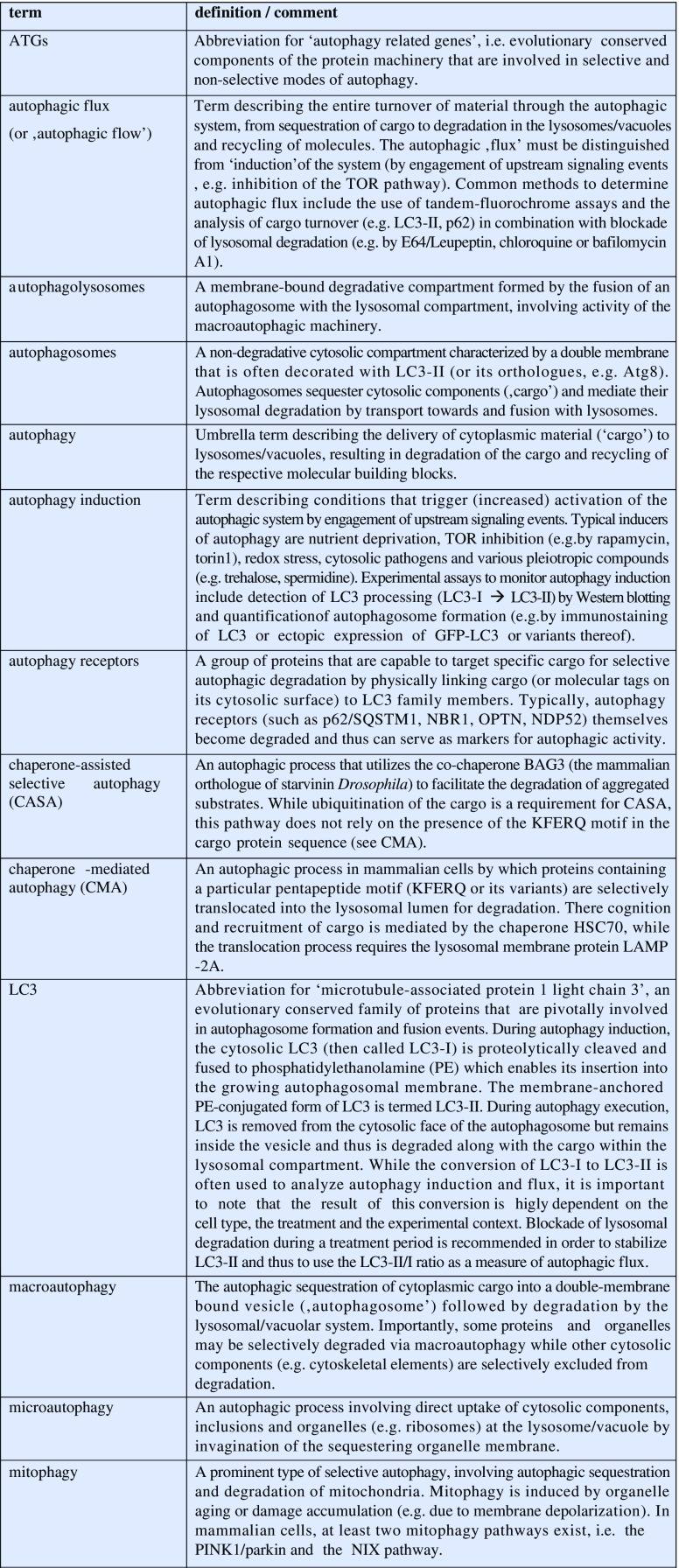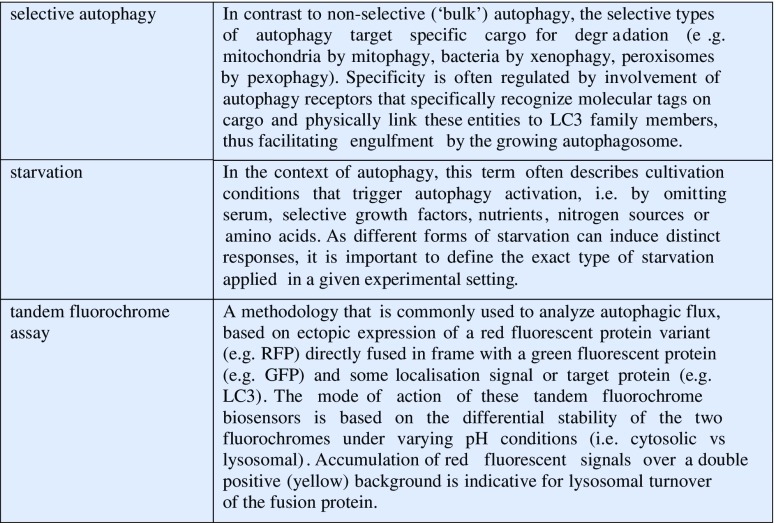# Induced pluripotent stem cell-based modeling of neurodegenerative diseases: a focus on autophagy

**DOI:** 10.1007/s00109-017-1533-5

**Published:** 2017-06-07

**Authors:** Johannes Jungverdorben, Andreas Till, Oliver Brüstle

**Affiliations:** 1 Institute of Reconstructive Neurobiology, University of Bonn Medical Faculty, Sigmund-Freud-Straße 25, 53105 Bonn, Germany; 2 Sloan-Kettering Institute for Cancer Research, 1275 York Avenue, New York, NY 10065 USA; 3 LIFE & BRAIN GmbH, University of Bonn, Sigmund-Freud-Strasse 25, 53105 Bonn, Germany

**Keywords:** Disease modeling, iPS cells, Neurodegenerative disease, Autophagy

## Abstract

The advent of cell reprogramming has enabled the generation of induced pluripotent stem cells (iPSCs) from patient skin fibroblasts or blood cells and their subsequent differentiation into tissue-specific cells, including neurons and glia. This approach can be used to recapitulate disease-specific phenotypes in classical cell culture paradigms and thus represents an invaluable asset for disease modeling and drug validation in the framework of personalized medicine. The autophagy pathway is a ubiquitous eukaryotic degradation and recycling system, which relies on lysosomal degradation of unwanted and potentially cytotoxic components. The relevance of autophagy in the pathogenesis of neurodegenerative diseases is underlined by the observation that disease-linked genetic variants of susceptibility factors frequently result in dysregulation of autophagic-lysosomal pathways. In particular, disrupted autophagy is implied in the accumulation of potentially neurotoxic products such as protein aggregates and their precursors and defective turnover of dysfunctional mitochondria. Here, we review the current state of iPSC-based assessment of autophagic dysfunction in the context of neurodegenerative disease modeling. The collected data show that iPSC technology is capable to reveal even subtle alterations in subcellular homeostatic processes, which form the molecular basis for disease manifestation.

## Introduction

“We have colonies.” and “We realized we had almost the entire pathway in our hands.” Two statements that changed the world of life sciences within less than a 15-year-timespan [1, 2].

The first statement refers to the colonies discovered by Kazutoshi Takahashi in Shinya Yamanaka’s small research group at Kyoto University in Japan back in 2006. The colonies were the result of 24 carefully chosen genes introduced via retroviruses into skin fibroblasts from mice, reprogramming them into what we now call induced pluripotent stem cells (iPSCs). In the weeks to come, the essential genes for the reprogramming process could be narrowed down to the four transcription factors Oct4, Sox2, Klf4, and c-Myc [3]. A few months later, this revolutionizing approach was successfully translated to human cells [4, 5].

**Fig. 1:**
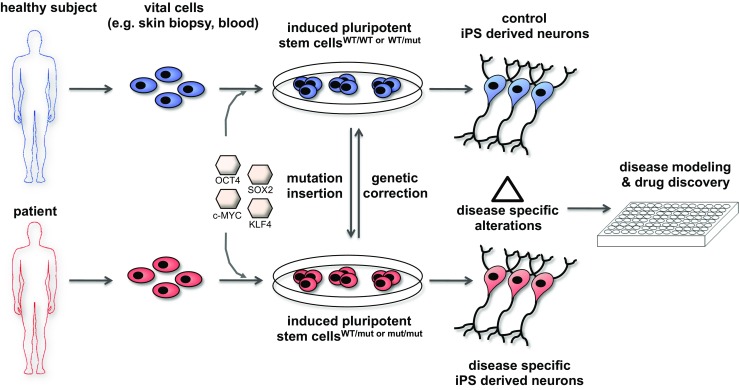
Generation of control and patient-specific iPSCs and their differentiation into neurons and glia for assessment of disease-specific alterations. Cells collected from healthy donors or affected patients (e.g., skin fibroblasts or blood cells) are reprogrammed to a pluripotent state by introduction of the transcription factors OCT4, SOX2, KLF4, and c-MYC (“Yamanaka factors”). Isogenic controls can be generated via genetic correction (e.g., genome editing) of the disease-related mutation. Conversely, disease-associated mutations can be inserted into iPSCs generated from healthy subjects to yield disease-specific iPSCs. Neural cells derived from control and disease-specific iPSCs are then used to decipher cellular and molecular alterations associated with the disease process (“disease modeling”). Neural cells generated this way may also be used for compound testing and drug discovery in a cell- and disease-specific human context

Pluripotent stem cells (PSCs) have the potential to differentiate into derivatives of all three germ layers, thus providing a route to generate any somatic cell type in limitless numbers in vitro. Unlike pluripotent embryonic stem cells, iPSCs can be derived from any patient or healthy donor, thereby opening unprecedented prospects for generating autologous donor cells for regenerative medicine, patient-specific disease models, and drug discovery (Fig. 1). In 2012, not even a decade after the first description of the first iPSCs, Shinya Yamanaka was awarded the Nobel Prize for this revolutionizing breakthrough together with Sir John B. Gurdon, who had shown reprogramming by nuclear transfer in *Xenopus* oocytes decades before [1].

**Fig. 2:**
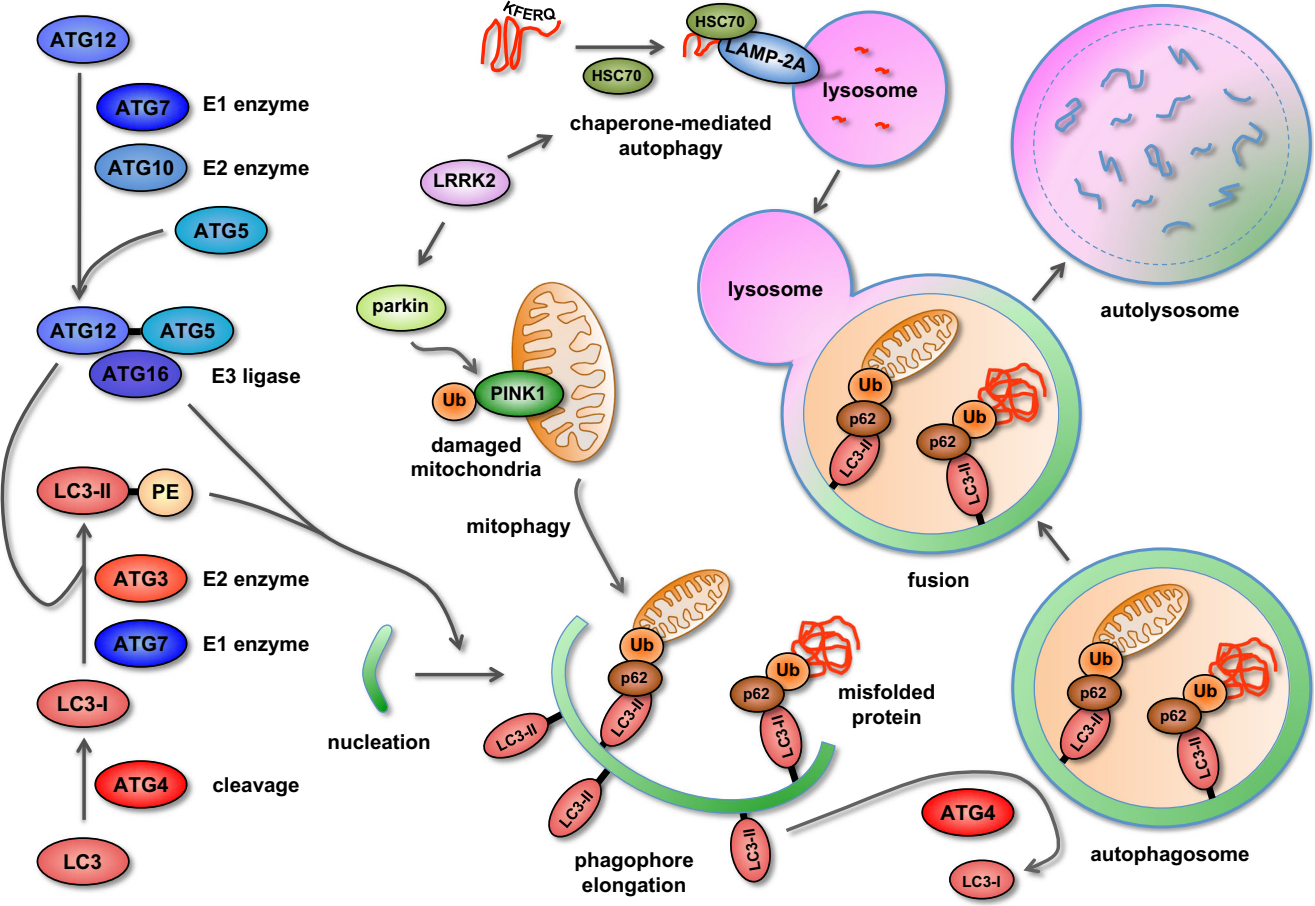
Regulation and execution of the autophagosomal-lysosomal degradation pathway. Two ubiquitin-like conjugation systems are involved in the regulation of autophagy. ATG12, a ubiquitin-like protein, is covalently bound to ATG5 by the E1 and E2 enzymes ATG7 and ATG10, respectively. In parallel, LC3 is cleaved by ATG4, primed by the E1 and E2 enzymes ATG7 and ATG3 to be covalently linked to the lipid phosphatidylethanolamine (PE) by the E3 ligase complex ATG12/5/16 to generate the processed, lipid-bound form of LC3, LC3-II. LC3-II and the ATG12/5/16 complex act together in the elongation and finally closure of the phagophore. LC3-II also acts as anchor on the inner membrane for adaptor proteins such as p62 that recognize ubiquitinated substrates, e.g., misfolded proteins or damaged organelles. On aged or dysfunctional mitochondria, PINK1 localizes to the outer membrane, where it is recognized and ubiquitinated by the E3 ubiquitin ligase parkin. Ubiquitinated PINK1 recruits autophagy receptors such as p62 and renders the mitochondria attractive for degradation by autophagy (“mitophagy”). After closure, the autophagosome with its cargo is primed for fusion with lysosomes, which is accompanied by removal and recycling of LC3-II by ATG4. The resulting autolysosome degrades the cargo and releases the components (amino acids, lipids) for metabolic and energy consuming processes. Substrates selected for lysosomal degradation by the signal sequence KFERQ can reach their destination also by binding to the chaperone HSC70 and LAMP2A-mediated direct import into the lysosomal lumen (chaperone-mediated autophagy; CMA). The kinase LRRK2 is involved in regulation of CMA and mitophagy, the latter via direct interaction with parkin. Ub: ubiquitin

**Fig. 3:**
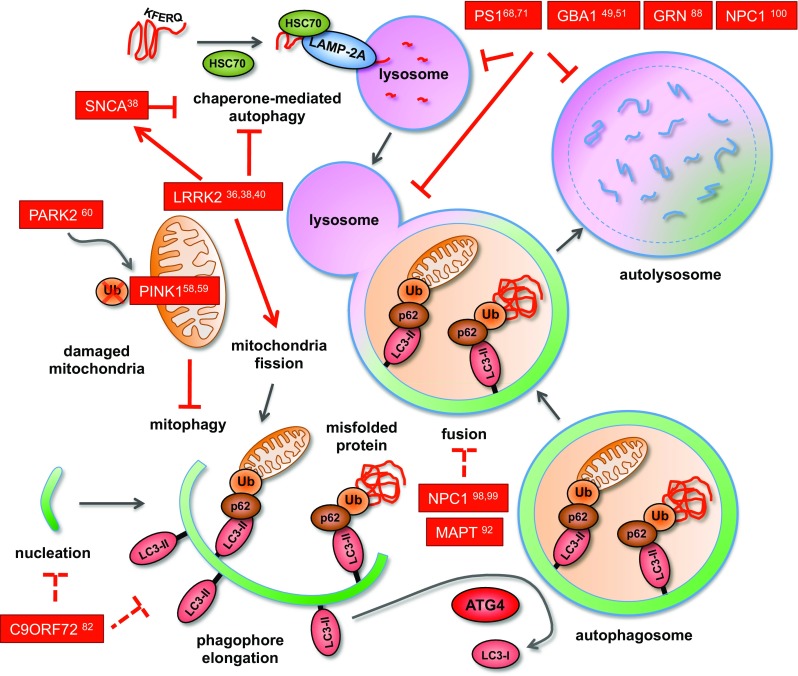
Modeling disease-associated autophagy in iPSC-derived neurons. Mutations studied in iPSC-derived neurons interfere with the autophagosomal-lysosomal degradation pathway at multiple levels. Mutations in C9ORF72 are associated with impaired phagophore nucleation and/or elongation. Fusion of autophagosomes and lysosomes is compromised by mutations in PS1, GBA1, GRN, and eventually NPC1 and MAPT. PS1, GBA1, and GRN mutations also cause direct defects in lysosomal function, thereby affecting autolysosomes as well as the CMA pathway. Mutations in parkin and PINK1 selectively impair mitophagy, probably by defective ubiquitination and/or impaired cargo recognition due to mutated binding sites. Mutated LRRK2 induces aberrant fission of mitochondria, thereby interfering with their degradation. LRRK2 mutants are also considered to interfere with CMA by elevating α-synuclein (SCNA) levels through mechanisms still to be elucidated. The numbers in superscript refer to the respective publications

The second statement refers to the discovery Yoshinori Ohsumi and his group in Tokyo made in 1993 when they discovered 15 genes in yeast that were essential for the autophagic process [6] which is induced in yeast mainly upon starvation. The term “autophagy” (from Greek αὐτό-/auto = „self-“and φαγεῖν/phageín = „eating”) was introduced by Christian de Duve in 1963 [7] as an umbrella term for the delivery of cytoplasmic material to lysosomes (or the vacuole in plants or fungi) for degradation. (For clarity on terminology used in this review, Textbox 1 provides an overview on autophagy-related terms, definitions, and methodology.) Today autophagy is subclassified into four variants: microautophagy [8], chaperone-assisted selective autophagy (CASA) [9], chaperone-mediated autophagy (CMA) [10, 11], and macroautophagy. The best-studied subclass in the mammalian system, macroautophagy (hereafter referred to as autophagy), is a process where a double membrane cup-shaped structure, the phagophore, forms to engulf a portion of the cytosol including protein aggregates, entire or parts of organelles and intracellular pathogens, and after closure delivers the cargo as mature autophagosome by fusion to lysosomes for degradation [12]. The genes discovered by Ohsumi and colleagues, nowadays called *Atgs* (*A* *u* *t* *opha* *g* *y related genes*), cover essentially the whole molecular machinery required for this process. In the years to follow, his group deciphered the precise mode-of-action of a large variety of previously uncharacterized *Atgs*, which turned out to form a ubiquitin-like conjugation and a lipidation system. Specifically, Atg12 was found to be a ubiquitin-like protein that is activated at its C-terminus by the E1 enzyme Atg7 and transferred to the E2 enzyme Atg10 before being covalently linked to Atg5 [13]. This Atg12-Atg5 conjugate, together with Atg16, then forms a complex essential for autophagy [14, 15], a process also conserved in mammalian cells [16, 17]. The second system for lipidation is composed of Atg8 as a precursor that is cleaved by the cystein protease Atg4, then activated and transferred by the E1 enzyme Atg7 to the E2 enzyme Atg3 and finally covalently bound to the lipid phosphatidylethanolamine (PE) [18]. Again, this system was found to be conserved in mammals, and the mammalian homolog of Atg8, LC3, is also cleaved by ATG4 to LC3-I and further processed by ATG7 and ATG3 to form lipidated LC3-II that is stably integrated into the membrane of the growing autophagosome [19] (Fig. 2). Detection of LC3-II provided the first reliable marker for mammalian autophagosomes, and its abundance correlates with autophagosome number [20]. The importance of autophagy in neuronal homeostasis was demonstrated only a few years later by Noboru Mizushima, a former postdoc of Ohsumi, and Komatsu et al. by CNS-specific conditional targeting of Atg5 and Atg7, respectively. Affected mice showed neurodegeneration, motor impairment, and formation of ubiquitin-positive intraneuronal protein inclusions [21, 22]. Since then, the role of defective autophagy as a key player in neurodegeneration [23] (Fig. 3), cancer [24], and aging [25] gained more and more attention. In 2016, Yoshinori Ohsumi was awarded the Nobel Prize for his seminal discovery.

In this review, we focus on the interface between these two Nobel Prize-awarded advances and present recent progress in using iPSCs for studying autophagy-related phenotypes in neurodegenerative disease.

### Parkinson’s disease

Parkinson’s disease (PD) is the second most common neurodegenerative disorder affecting ∼2% of the population over the age of 60 [26]. Prominent symptoms of PD include motor deficits like tremor, bradykinesia, and limb rigidity. The neuropathology underlying these symptoms is a progressive preferential loss of ventral dopaminergic (DA) neurons in the *pars compacta* of the *substantia nigra*. A histopathological hallmark is the occurrence of intra-cytoplasmic ubiquitin-positive inclusions in surviving neurons known as “Lewy bodies,” which are mainly composed of the neuronal protein α-synuclein [27]. Approximately 90% of all PD cases are sporadic with no family history but there are several inherited or de novo mutations that cause PD [28, 29]. For most of them, patient samples have already been used to generate iPSC-derived neurons. Here, we will present the results regarding autophagy and PD chronologically in the background of the inherited mutation.

#### LRRK2

PD-causing mutations in the leucine-rich repeat kinase 2 *(LRRK2)* are autosomal dominant and manifest as a late-onset PD that is clinically and pathologically indistinguishable from the common idiopathic form [30, 31]. For that reason, *LRRK2* mutations represent an interesting target for iPSC-based research into the pathomechanisms underlying PD. While there are over 50 different variants involving various *LRRK2* domains in PD patients, the G2019S mutation is the most prevalent one, being detectable in up to 2% of sporadic PD cases [32]. Interestingly, an increased kinase activity of LRRK2 has been proposed to mediate the neurotoxic effect of this mutation [33].

In the study from Sanchez-Danes and colleagues [34], iPSCs from 4 *LRRK2* ^*G2019S*^ PD patients, 7 patients with sporadic (non-LRRK2^G2019S^-related) PD, and 4 healthy control donors were generated. The differentiation into midbrain dopamine (DA) neurons was achieved by overexpression of LMX1A in a 30-day protocol [35], and no significant difference in the yield of neurons was detected, although the fraction of DA neurons was only 9–29%. Analysis of LC3 in the neuronal cultures by immunoblot showed increased LC3-II levels in PD samples compared to controls, and inhibition of autophagosome-lysosome fusion [20] revealed an impairment of autophagic flux in all PD lines. In addition, electron microscopy (EM) showed accumulation of lipid droplets as well as more autophagosomes compared to autophagolysosomes in PD lines, indicative for a defect in autophagic flux through inhibited fusion of autophagosomes with lysosomes.

Su and Qi found that *LRRK2* ^*G2019S*^ DA neurons exhibit excessive autophagic and lysosomal activity, and mitochondrial dysfunction compared to control DA neurons could be detected at day 30 of differentiation in a protocol based on patterning cues for the ventral midbrain [36]. Interestingly, these phenotypes could be partially reverted by inhibition of mitochondrial fission, pointing to a highly intertwined functional crosstalk between mitochondrial function and autophagy.

Orenstein and colleagues analyzed whether chaperone-mediated autophagy (CMA) in *LRRK2* ^*G2019S*^ DA neurons is compromised by the interaction of α-synuclein with lysosome-associated membrane protein type 2A (LAMP-2A), the essential component for CMA [10, 37]. They found elevated α-synuclein levels in 30-day-old DA neurons from two *LRRK2* ^*G2019S*^ lines compared to two controls and reported a dramatically increased co-localization of α-synuclein with LAMP-2A [38]. As α-synuclein is considered a substrate of CMA by shuttling through the lysosomal channel protein LAMP-2A into the lysosome, these findings suggest impaired activity of the CMA pathway in this experimental system [38]. Interestingly, the increase in the number of α-synuclein^+^ DA neurons between day 30 and day 75 in culture could be further increased by knockdown of LAMP-2A.

Reinhardt and colleagues generated isogenic *LRRK2* ^*G2019S*^ iPSCs from a healthy control line and compared both lines with respect to their LC3-II flux. In this system, a reduced autophagic flux could be detected in the engineered PD line under starvation conditions [39].

Very recently, light was shed on the mechanism how the LRRK2^G2019^ mutation affects the turnover of damaged mitochondria [40]. Hsieh et al. could show that in *LRRK2* ^*G2019*^ iPSC-derived DA neurons, the mitochondrial protein Miro persisted longer on the outer membrane of mitochondria after treatment with the complex III inhibitor antimycin A than on control DA neurons. This was accompanied by delayed recruitment of LC3 and the autophagy receptor optineurin to damaged mitochondria, further arguing for an impairment of mitophagy. In addition, a failure in LC3 recruitment and a delayed turnover of damaged mitochondria could be detected in DA neurons of two sporadic PD lines, thereby implicating a general role of impaired mitophagy in PD.

Ohta et al. investigated another PD-causing *LRRK2* mutation, I2020T, located in the LRRK2 kinase domain, using iPSC-derived neurons [41]. They discovered slightly increased levels of p62/SQSTM1, a generic autophagy substrate [42], and elevated levels of LC3-II in *LRRK2* ^I2020T^ neuronal cultures compared to controls.

Taken together, the data from PD iPSC models relating to autophagy and LRRK2 mutations point to a model where defective autophagosome-lysosome fusion and delayed turnover of damaged mitochondria might conspire with impaired CMA, thus favoring α-synuclein accumulation, increased sensitivity to oxidative stress, and eventually demise of DA neurons.

#### GBA1

In recent years, heterozygous mutations in the ß-glucocerebrosidase (*GBA1*) gene, which encodes the lysosomal enzyme ß-glucocerebrosidase (GCase), have been associated with a higher risk of developing PD, thereby challenging the role of *LRRK2* mutations as most common monogenic risk factors for PD [43–45]. GCase is a lysosomal enzyme that hydrolyzes glucosylceramide (Glc-Cer) to ceramide and glucose. Homozygous mutations of *GBA1* are causing Gaucher’s disease (GD), the most prevalent lysosomal storage disorder [46]. GD patients have a 20-fold increased lifetime risk for developing PD [47]; for heterozygous *GBA1* carriers, the lifetime risk is still five times higher than in the general population [43]. Considering these numbers and the fact that *GBA1* mutations are also associated with Lewy body dementia [48], understanding the downstream disease mechanisms resulting from these mutations is of great importance.

Exploiting the reprogramming technology, Schöndorf and colleagues generated two control iPSC lines, four *GBA1* PD lines, and two GD lines, and even genetically corrected two of the *GBA1* PD lines [49]. In both *GBA1* PD and GD, there was an accumulation of LAMP1, a lysosomal marker, in DA neurons at day 65, which was reverted to control levels in gene-corrected cells. In addition, LC3 and LAMP1 co-localized less in *GBA1* mutant DA neurons, and LC3-II levels where elevated compared to control and gene-corrected cells. Assessment of autophagic flux revealed impairment of autophagosome-lysosome fusion in *GBA1* DA cultures that was not present in control cells and reduced in gene-corrected cells. The authors also reported dysregulated calcium signaling (a mechanism which may contribute to autophagy impairment [50]) and elevated α-synuclein as well as Glc-Cer levels that conform to the histopathological phenotype in patient brains.

More recently, Fernandes et al. made use of three control and three unrelated *GBA1* PD iPSC lines to generate DA neurons and analyzed them at day 31–35. Their data revealed elevated levels of LC3-II, p62, LAMP1, LAMP-2A, and beclin-1 in *GBA1* PD DA neurons compared to controls. These observations strongly suggest that autophagosomal-lysosomal turnover is impaired in the mutant lines, although autophagic flux was not directly investigated [51]. Strikingly, EM analysis confirmed an increased number of autophagosomes and lysosomes with undigested cargo. Taken together, the experimental evidence emerging from these studies points to an impaired autophagic flux as a contributing factor for *GBA1*-linked PD.

#### PINK1 and parkin/PARK2

PTEN-induced putative kinase 1 (*PINK1*) and parkin (*PARK2*) exert essential functions in mitochondrial homeostasis and quality control [52–54]. Recessively inherited mutations in both genes are related to PD [55, 56]. Parkin functions as an E3 ubiquitin ligase that ubiquitinates PINK1 on the outer membrane of damaged and aged mitochondria [54, 55] to enable their removal by p62-mediated autophagy, a process referred to as “mitophagy” [57].

For the investigation of PINK1, Seibler et al. generated one iPSC line each from one *PINK1* PD patient and one healthy family member, derived DA neurons and, at day 60 of differentiation, analyzed the recruitment of parkin to mitochondria upon treatment with the potassium carrier and antibiotic valinomycin [58]. They observed that *PINK1* PD DA neurons—in contrast to controls—showed no recruitment of parkin to mitochondria. Furthermore, no reduction of mitochondrial DNA, an indicator of damaged mitochondria removal, could be detected. Both defects, lack of parkin recruitment and impaired mitochondria removal, were restored by overexpression of wild-type PINK1. This phenotype of defective parkin recruitment to mitochondria in *PINK1* PD DA neurons upon valinomycin treatment could be recapitulated by Rakovic and colleagues using one *PINK1* PD iPSC line and one control [59].

To investigate the effect of parkin mutations, Shatoulki et al. used 4-week-old DA neurons derived from four *PARK2* PD patients, one healthy control along with hetero- and homozygous *PARK2* knockout iPSC lines [60]. Although parkin localization or direct mitophagy was not assessed, all parkin-deficient lines, hetero- and homozygous, generated less DA neurons, with *PARK2* patient neurons containing less mitochondrial mass than control neurons. Collectively, these data suggest that impairment of mitochondria turnover is a contributing factor in PINK1/parkin-related PD.

### Alzheimer’s disease

Alzheimer’s disease (AD) is the most frequent form of dementia and the most common neurodegenerative disorder. AD is clinically characterized by progressive loss of memory and neuropathologically by the presence of amyloid plaques and neurofibrillary tangles [61]. Intriguingly, functional abnormalities of autophagosomes and lysosomes were found to precede these paradigmatic pathological changes in AD brains [62]. Interestingly, presenilin 1 (PS1), the most commonly affected gene in early onset familial AD [63], is essential for lysosomal and autophagy function [64] with AD-linked *PS1* mutations impairing these pathways [65]. In addition, elevated levels of acid sphingomyelinase (ASM) are associated with AD [66], and sphingomyelin metabolism is regulated by presenilins [67]. Alterations of ASM activity could therefore be a downstream effect of presenilin mutations in AD.

Addressing this relationship, Lee and colleagues reprogrammed cells from one *PS1* AD patient and one control subject to iPSC and assessed the ASM levels in the clonally derived *PS1* lines and their neuronal derivatives [68]. In the *PS1* iPSC line with the highest ASM levels, LC3-II and p62 levels in iPSC-derived neurons were elevated whereas the levels of LAMP1 and the basic helix–loop–helix transcription factor EB (TFEB), a transcriptional master regulator of autophagy [69, 70], were decreased. EM analysis of iPSC-derived neurons revealed an accumulation of autophagosomes in *PS1* AD neurons. Knockdown of ASM by siRNA in *PS1* AD neurons normalized the levels of LC3-II, p62, LAMP1, and TFEB comparable to control neurons, reduced autophagosome number detected by EM, and further partially restored the impaired expression of TFEB target genes such as cathepsin B. These findings argue for a defect in autophagosomal-lysosomal fusion mediated by ASM activity in *PS1* AD neurons.

Using two PS1 AD and two control iPSC lines, Reddy et al. found that *PS1* AD neurons exhibit decreased nuclear calcium signaling compared to control neurons [71]. Moreover, knockdown of PS1, cAMP responsive element binding protein (CREB) or calcium/calmodulin dependent protein kinase IV (CaMKIV) in control neurons resulted in decreased expression of sestrin2, LC3 and p62. Noteworthy, PS1 overexpression could not rescue the observed gene expression alterations in the CREB/CaMKIV knock-down setting, thus placing dysregulated nuclear calcium signaling downstream of the *PS1* mutation. Upon application of a calcium ionophore increasing the cytosolic calcium level, LC3-GFP punctae were increased in *PS1* AD neurons; in the same setting, PS1 depleted neurons showed a calcium dependent increase of sestrin2 with the decreased autophagic flux being reestablished. Taken together, these findings suggest that *PS1* AD iPSC-derived neurons show a deficiency in autophagic flux, which is probably mediated by altered nuclear calcium signaling as well as altered sphingomyelin metabolism and subsequent downregulation of TFEB and its target genes.

### Frontotemporal dementia/amyotrophic lateral sclerosis

The neurodegenerative disorder manifesting clinically as frontotemporal dementia (FTD) is genetically a pleiotropic group of sporadic cases and identified different mutations [72, 73]. Some of the mutations linked to FTD also manifest as amyotrophic lateral sclerosis (ALS) [74]. FTD is characterized by focal but progressive neuronal atrophy in the frontal and temporal cortices as well as astrogliosis, inflammation, and prominent intracellular protein inclusions, mostly positive for ubiquitin, tau protein, p62, and TAR DNA-binding protein 43 (TDP43) [75, 76]. Given the heterogeneity of FTD, this section will focus on distinct genetic changes that have been addressed using iPSC models.

#### C9ORF72

The hexanucleotide repeat expansion GGGGCC in the non-coding region of the *C9ORF72* gene is the most common known pathogenic mutation underlying FTD and ALS [77–79]. A number of different pathogenic mechanisms have been proposed, including C9ORF72 haploinsufficiency, RNA toxicity, and repeat-associated non-ATG (RAN) translation of neurotoxic dipeptides [80, 81].

In their search for neuropathological phenotypes in *C9ORF72* iPSC-derived neurons from two patients compared to one control, Almeida and colleagues stressed the neuronal cultures around day 35 with the lysosomal inhibitor chloroquine and the autophagosome formation inhibitor 3-methyladenine [82]. C9ORF72 neuronal cultures displayed a higher vulnerability and increased cell death upon inhibitor exposure than control cultures and showed increased levels of p62, although autophagic flux was not assessed. These findings could point to a contribution of autophagic impairment to the pathogenesis of *C9ORF72*-related FTD and also ALS. Interestingly, a recent study established a link between loss of *C9ORF72* and impairment of autophagy, as C9ORF72 is involved in regulating autophagy induction via forming a complex with SMCR8 and WDR41, two proteins involved in the early steps of autophagosome formation [83].

#### GRN

Progranulin (PGRN) is a secreted glycoprotein involved in cell survival, inflammation, and neuroprotection [84, 85]. In the vast majority of cases mutations in the *GRN* gene encoding PGRN result in haploinsufficiency and decreased expression of the protein. The complete loss of PGRN is one genetic cause for early-onset neuronal ceroid lipofuscinosis, a pleiotropic multiform lysosomal storage disorder, implicating a role in lysosome homeostasis [86].

To treat the haploinsufficiency of PGRN, Holler et al. screened for potential inducers of PGRN expression and went on to validate their hit, the autophagy inducer trehalose [87], in 25-day-old iPSC-derived motor neurons generated from one control and one patient-derived GRN line [88]. Application of trehalose elevated PGRN and LC3-II in GRN neurons, but there was no increase of LC3-II in control neurons. Unfortunately, autophagic flux was not assessed in this study, which will be required to determine whether trehalose represents a therapeutic option for treating PGRN- associated FTD.

#### MAPT

The tau protein, encoded by the *MAPT* gene, is ubiquitously expressed in the brain with a predominant localization in axons, where it is involved in microtubule polymerization and organelle transport [89]. There are many different mutations in *MAPT* linked to FTD [87], including the A152T mutation [90, 91].

Characterizing their iPSC-based FTD model comprising two patient lines, two control lines and also a *MAPT* knockout line, Silva et al. discovered elevated levels of LC3-II, p62, LAMP1, LAMP-2A, ATG12-5 and also an accumulation of ubiquitinated proteins in 35-day-old cortical neuronal cultures derived from *MAPT* ^*A152T*^ iPSCs [92]. Interestingly, the detected susceptibility to rotenone, NMDA or amyloid-ß toxicity as well as elevated levels of tau and phospho-tau could be attenuated by treatment with rapamycin. Although autophagic flux was not directly investigated, the observed reduction of tau protein, a known substrate of autophagy [93], could suggest that the effect is elicited by an increase in autophagic flux, thereby depicting rapamycin as potential candidate for the treatment of *MAPT*-linked FTD.

### Niemann-Pick type C disease

Like GD, Niemann-Pick type C disease (NPC) is an inherited autosomal recessive lysosomal storage disorder where >95% of cases are caused by loss-of-function mutations in the *NPC1* gene, leading to severe neurodegeneration and liver dysfunction [94, 95]. The result of lost NPC1 function is impaired cholesterol homeostasis that mediates damage in liver and brain [96]. Interestingly, autophagy is involved in lipid metabolism [96] and, conversely, cellular lipid content affects autophagosomal membrane turnover [97]. Therefore, alterations in lipid composition are likely to affect the autophagy pathway.

As NPC is a recessive disorder, Maetzel et al. reprogrammed four NPC patients, one heterozygous healthy control and one homozygous control, and even genetically corrected one NPC line to a homozygous wild type genotype [98]. Neuronal cultures from NPC patients showed elevated LC3-II and p62 levels compared to control cells and the isogenic control at day 28, and the autophagic flux in NPC neurons was reduced. A compound screen for normalization of p62 levels revealed carbamazepine as a potential drug for NPC treatment.

Using one NPC iPSC line and one healthy control, Lee and colleagues detected also elevated LC3-II and p62 levels in NPC neurons compared to control cultures. In addition, they assessed autophagosome-lysosome turnover by electron microscopy and a fluorescence mCherry-eGFP-LC3 reporter assay (where eGFP fluorescence is quenched in the acidic lysosomal environment, thereby labeling fused autolysosomes purely red [99]). Both EM and LC3 reporter analysis revealed an accumulation of autophagosomes in NPC neurons compared to control cells. The detected autophagosomal-lysosomal turnover impairment could be rescued by application of vascular endothelial growth factor (VEGF).

The third study that reported an elevation of LC3-II and p62 in NPC iPSC-derived neural cells compared to control cultures was performed by Soga et al. using two NPC iPSC lines (two clones each) and two control iPSC lines [100], though only neural progenitor cells were analyzed. Nevertheless, treatment of NPC neural progenitors with 2-hydroxypropyl-c-cyclodextrin, a substance that induces release of cholesterol from late endosomes and lysosomes [98], decreased LC3-II and p62 levels without effecting viability. All three NPC iPSC-based studies presented here point to a reduced autophagic flux as pathogenic mechanism underlying NPC. They further exemplify the potential usefulness for iPSC-derived neurons for validating drug candidates that may restore autophagic flux.

## Appropriate controls and cell types matter

A closer look at studies with different patient lines reveals that a disease-specific phenotype can vary markedly amongst iPSC lines derived from different subjects and even between clones generated from the same subject, although there might still by a significant difference to control lines [68]. To avoid false conclusions in either direction that are solely based on genetic variability and not disease-related, the generation of isogenic iPSC pairs is strongly recommended whenever possible. Generating isogenic iPSC lines is greatly assisted by emerging genome editing techniques such as the CRISPR/Cas9 system [101].

One of the major puzzling questions associated with the pathogenesis of neurodegenerative diseases is why ubiquitously expressed mutant proteins exert their main detrimental effects specifically in neurons and even in defined neuronal subtypes. This basic observation calls for model systems which enable pathogenetic studies in distinct human neuronal subtypes, a scenario which has become palpable with the advent of cell reprogramming and recent progress in the in vitro differentiation of iPSC towards a number of neural sublineages. Indeed, some of the phenotypes observed in the PD-related iPSC studies discussed in this review were reported to be visible only when DA neurons were assessed [38, 51, 58]. Along the same line, in a recently reported ALS iPSC study, a neurofilament phenotype could only been delineated in motoneurons but not in interneurons [102]. Thus, choosing the right differentiation protocol and neuronal subtype for each disease can be of major importance, although it might be possible to discover disease-related alterations in other neural subtypes as well.

## Flux or no flux

Frequently, controversies in the field of autophagy arise from the different interpretation of observed changes as either activation or inhibition of autophagy. This phenomenon underscores the necessity to differentiate between the induction of the autophagic process and the concept of autophagic flux [20]. For example, elevated levels of LC3-II can result either from increased induction of autophagic activity (e.g., by inhibition of the mTOR pathway) or by blockade of autophagic flux via impairment of lysosomal degradation [20]. The majority of the studies revisited in this review took this caveat already into consideration, but some results could still faithfully be interpreted the opposite way the authors initially suggested. To avoid such ambiguity, future studies should assess autophagic flux whenever possible, e.g., by introduction of tandem fluorochrome reporter systems for measurement of direct turn-over of autophagy substrates, or by the use of lysosomal inhibitors (such as Bafilomycin A, or the combination Leupeptin/E64) to block the endpoint of degradation. In addition, the investigation of additional parameters such as p62 or BECN1 turnover in addition to analysis of LC3 conversion is recommended. It is important to realize that such studies can still be confounded by, e.g., context-dependent transcriptional (up-) regulation of LC3 and p62, or spontaneous aggregation of reporter fusion proteins such as p62-GFP [103, 104].

## Outlook

While autophagy and iPS cell-based disease modeling have both become key topics in neurodegeneration research (see summary in Table 1), there is still a lot of room for synergyzing these two fields. The studies presented here show that iPSC models can be used successfully to identify autophagy-related phenotypes associated with different neurodegenerative diseases. This approach is not only suitable for the detection of new autophagy-related pathomechanisms but also for corroborating or dismissing presumptive disease-related disturbances in autophagy described in other, less authentic model systems.

**Table 1:** iPSC-based models of neurodegenerative diseases with focus on the autophagy-lysosomal system

Disease	Disease-linked gene	Cell type analyzed	No. of patient/control lines	Proposed autophagy-related pathophenotype	Reference
PD	LRRK2 (G2019S)	Midbrain DA neurons	11/4^a^	Defective autophagosome clearance	[35]
	LRRK2 (G2019S)	DA neurons	1/1	Excessive mitochondrial fission ➔ exacerbated autophagy induction	[36]
	LRRK2 (G2019S)	DA neurons	1/2	LRRK2^G2019S^-dependent impairment of CMA	[38]
	LRRK2 (G2019S)	Neurons	1/1^b^	Autophagic defect under starvation conditions	[39]
	LRRK2 (G2019S)	DA neurons	3/3^c^	Impaired mitochondria turn-over by Miro stabilization	[40]
	LRRK2 (I2020T)	Neurons	2/2	Decreased autophagic flux	[41]
	GBA1	DA neurons	4/4^d^	Impaired autophagosome-lysosome fusion	[49]
	GBA1	DA neurons	3/3	Impaired lysosomal degradation	[51]
	PINK1	DA neurons	1/1	Defective Parkin-mediated mitophagy	[58]
	PINK1	DA neurons	1/1	Defective Parkin-mediated mitophagy	[59]
	PARK2	DA neurons	5/1^e^	Defective mitophagy	[60]
AD	PS1	iPSCs and neurons	1/1	Lysosomal depletion and defective autophagic degradation	[68]
	PS1	Neurons	2/2	Impaired autophagy	[71]
FTD	GRN	Motor neurons	1/1	Impaired autophagosomal-lysosomal turnover	[88]
	MAPT	Cortical neurons	3/2^f^	Impaired autophagosomal-lysosomal turnover	[92]
ALS & FTD	C9ORF72 ^g^	Neurons	2/1	Impaired autophagy	[82]
NPC	NPC1	Neurons	4/3^h^	Impaired autophagic flux	[98]
	NPC1	Neurons	1/1	Impaired autophagosomal-lysosomal turnover	[99]
	NPC1	Neural progenitors	2/2	Impaired autophagy	[100]

^a^7 sporadic PD + 4 LRRK2-mut

^b^Engineered/parental

^c^3 controls (2 healthy +1 gene corrected)

^d^4 controls (2 healthy +2 gene corrected)

^e^4 iPSC disease lines +1 PARK2—knock out line/1 control

^f^2 iPSC disease lines +1 MAPT—knock out line/1 control

^g^C9ORF72 (polyGGGGCC)

^h^3 controls (2 healthy +1 gene corrected)

Of particular interest will be the further investigation of iPSC models of diseases where a pathologically aggregating protein is a known autophagy substrate such as, e.g., in the polyglutamine (polyQ) disorders Spinal and Bulbar Muscular Atrophy (SBMA) [105], Huntington’s Disease (HD) [106], and Machado-Joseph Disease (MJD) [107]. Experiments in this direction are bound to provide more insight into the pathogenic role of autophagy as exemplified by a recent study showing impairment of TFEB-mediated autophagy already in iPSC-derived neural precursors derived from SBMA patients [105]. Such an early manifestation of a phenotype raises the question whether autophagy impairment exerts its pathogenic role already during development.

Further key questions to be addressed are whether and to what extent autophagy is differently regulated in neuronal subtypes and why general modulation of autophagy elicits specific effects in individual cellular subtypes [108, 109]. IPSC-derived neurons could also be used to gain insight into the role of autophagy during age-associated progression of a neurodegenerative phenotype [110] and potentially open new avenues towards slowing down the aging process by precise modulation of autophagic pathways [25].

Future studies may also address a potential disease-related role of autophagy in glial cells. While autophagy appears to be particularly relevant in neurons due to the unique biology of this cell type (postmitotic state, high energy demands, extreme life span), there is growing evidence for specialized functions of autophagy pathways in glial cells (reviewed in [111]). Examples include a contribution to myelination in oligodendrocytes, a possible role in synaptic pruning in astrocytes, and autophagy-mediated cell death in microglia [111]. Furthermore, there is evidence that oligodendroglial α-synuclein inclusions in multiple system atrophy (MSA) patients are co-localizing with LC3, p62 and ubiquitin [112, 113]. It will be interesting to decipher the role of autophagy in the generation of these glial inclusions using iPSC-derived oligodendrocytes and whether a dysregulation of autophagy contributes to the pathogenesis of MSA and other neurological disorders.

Taken together, while both, cell reprogramming and autophagy, have already been subject of Nobel Prizes, the amalgamation of both research lines towards understanding and treating neurodegenerative disorders has just begun.

Textbox 1. Definition of autophagy-related terms.